# Titin Cardiomyopathy, Emerging Evidence: More Than A Big Heart

**DOI:** 10.1007/s11886-025-02309-5

**Published:** 2025-12-02

**Authors:** Federico Angriman, Francesca Bortolotti, Maria Perotto, Rebecca Artioli, Cinzia Radesich, Alessia Paldino, Chiara Collesi, Serena Zacchigna, Gianfranco Sinagra, Matteo Dal Ferro

**Affiliations:** 1 Cardiothoracovascular Department, Azienda Sanitaria Universitaria Giuliano Isontina (ASUGI), Trieste, Italy; 2 Department of Medical, Surgical and Health Sciences, University of Trieste, Trieste, Italy; 3 International Centre for Genetic Engineering and Biotechnology (ICGEB), Trieste, Italy; 4 Department of Life Sciences, University of Trieste, Trieste, Italy; 5 Centro Cardiovascolare, Azienda Sanitaria Universitaria Giuliano Isontina (ASUGI), Trieste, Italy

**Keywords:** TTN-cardiomyopathy, Titin bands, Truncating variants, Haploinsufficiency/dominant negative effect, Reverse remodeling, Red flags, Risk stratification

## Abstract

**Purpose of Review:**

Titin-related cardiomyopathy (TTN CMP) is the most prevalent genetic cause of dilated cardiomyopathy (DCM), yet its clinical variability and incomplete penetrance challenge risk stratification and therapeutic management. This review aims to summarize recent insights into the genetic, molecular, and clinical aspects of TTN-CMP, emphasizing its heterogeneity and the implications for diagnosis, prognosis, and treatment.

**Recent Findings:**

*TTN* truncating variants (*TTN*tv) are associated with DCM but are also present in asymptomatic individuals, complicating their interpretation. Emerging evidence suggests that both haploinsufficiency and toxic gain-of-function mechanisms may underlie disease expression, influenced by variant location and expression. Patient-derived iPSC models and advanced imaging studies have elucidated pathophysiological mechanisms and highlighted promising therapeutic targets. Clinically, TTN-CMP shows relatively high rates of left ventricular reverse remodeling and variable arrhythmic burden, with male sex and environmental stressors influencing outcomes.

**Summary:**

TTN-CMP spans a broad phenotypic spectrum, ranging from asymptomatic carriers to end-stage heart failure. While standard heart failure therapies are often effective, precision management remains limited. Improved clinical, genetics and molecular understanding, together with novel experimental models, provide promising new tools for precise disease assessment and targeted therapeutic interventions.

## Introduction

Cardiomyopathies have emerged as prevalent conditions over recent decades, with a significant impact on both morbidity and mortality of the general population [1].

Increasing evidence supporting a genetic aetiology of these diseases has gradually shifted the focus from conventional phenotypic classifications to a gene-based approach, which has proven more effective in predicting disease development and outcomes [1, 2]. Among the genes implicated in non-hypertrophic cardiomyopathies, *Titin* has consistently been identified as the most common one [1, 3]. 

Titin, the largest protein encoded by the human genome, is fundamental to sarcomere organization providing structural stability and elasticity. It also plays a key role in mechanosensing and signaling pathways that regulate heart muscle function and response to stress [4]. Truncating variants of the *Titin* gene (*TTN*tv) are strongly associated with dilated cardiomyopathy. Instead, due to the large size of the gene, *Titin* non truncating variants (*TTNntv*) are frequently found in the population and are not usually linked to the development of cardiac disease [5]. 

*TTN*tv primarily affect the structural role of the protein, via haploinsufficiency and dominant negative effects [6]; these mechanisms impair sarcomere integrity and compromise the contractile capacity of the cardiomyocyte. However, recent advancements in basic science have unveiled additional molecular roles of Titin beyond sarcomere stability and contraction, particularly highlighting its involvement in cellular signalling [7].

Despite the presence of a similar genetic background, the clinical presentation, arrhythmic burden, and therapeutic response in Titin cardiomyopathies remain highly heterogeneous [8]. Risk stratification remains challenging, especially in the early stages of the disease, while early intervention and identification has currently provided limited benefits.

But what do we know and what are we still missing on Titin cardiomyopathy?

**Fig. 1:**
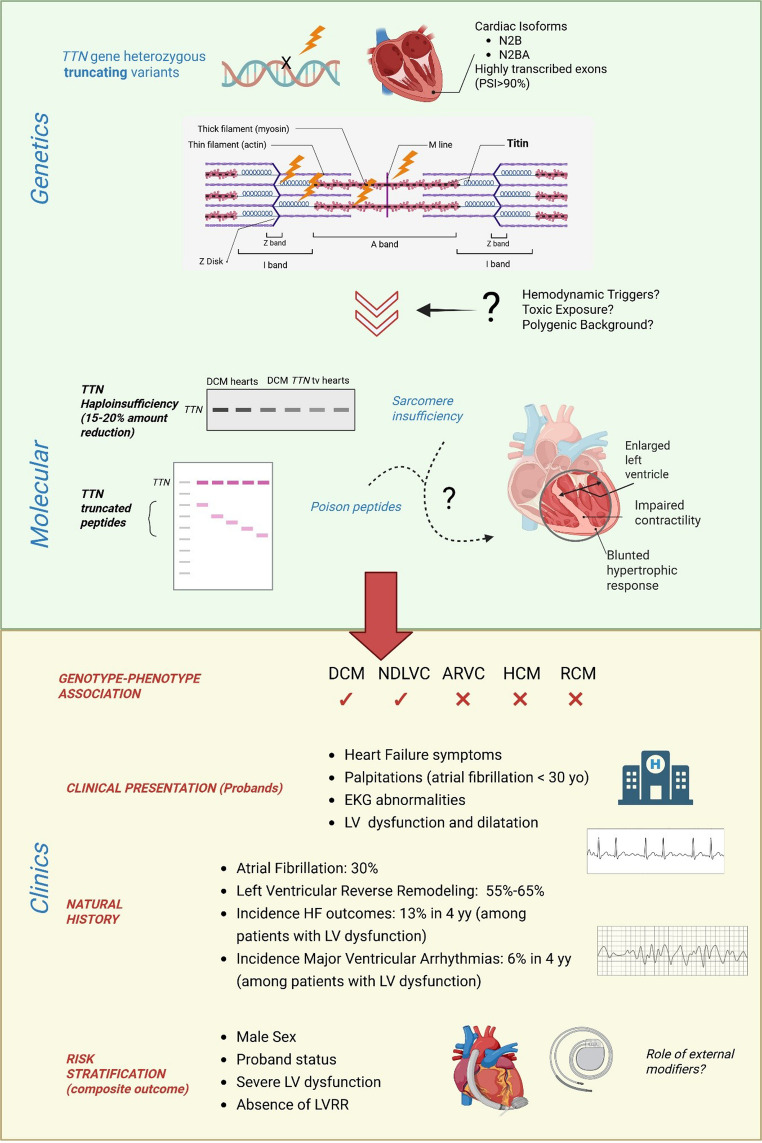
Genetics, molecular, and clinical aspects of TTN-CMP. *Figure created in BioRender. Guarnaccia, A. (2025)* https://BioRender.com/c8f00kb

This review will provide a comprehensive overview of the genetic and molecular basis of *TTN*-induced cardiomyopathy (*TTN*-CMP), highlighting recent discoveries and their translational implications. Additionally, we will offer an in-depth clinical summary focused on the prognosis and management of *TTN*-CMP based on current knowledge (Fig. 1).

## Genetics

The human *TTN* gene, located on the long arm of chromosome 2 at locus 2q31, encompasses 364 exons. This vast gene architecture provides extensive potential for alternative splicing, allowing for the creation of different protein isoforms. Isoforms vary throughout development and across different tissues. In the heart, the most prevalent isoforms are N2BA (NM_001256850.1) and N2B (NM_003319.4), which mostly differ in terms of stiffness properties and whose relative proportion changes in physiological and pathological conditions. The shorter and stiffer N2B isoform plays the major role in normal adult left ventricle. The N2A isoform (NM_133378.4), instead, is expressed in skeletal muscles and is associated with some rare forms of dystrophy [9]. In addition to full-length titin molecules, smaller isoforms such as Cronos and Novex 1–3 have recently been identified: to date, their role is still under investigation, both in developmental processes and in pathogenesis of cardiac disease [10]. 

Each titin protein spans one-half of the sarcomere extending from the Z-disk to the M-band and therefore it can be divided into four protein domains, moving from the C-terminal to the N-terminal: Z-disk, I-band, A-band and M-band [11–13]. 

A measure of whether an exon is incorporated into the cardiac mRNA transcript of *TTN* is provided by the PSI (Percentage Spliced-In), which estimates the percentage of *TTN* transcripts that incorporate that given exon derived from RNA sequencing studies in left ventricular tissue. When PSI is higher than 90% for the adult N2B isoform, variants affecting those exons are considered fully penetrant [14]. 

(Notably, *TTN* alternative splicing is regulated also by splicing factor RBM20. The interaction between deregulated RBM20 and TTN represents a potential mechanism of cardiac disease [15, 16]).

Variants in the *TTN* gene can be grouped in *TTNtv* and *TTNntv*. This classification is crucial for understanding their potential impact on protein function and disease development. *TTN*tv include all those variants that produce a truncated titin protein when they are transcribed into mRNA and translated into protein (nonsense, frameshift insertions/deletions, splice-site variants that disrupt a canonical splice-site). *TTNntvs* instead are missense variants (single nucleotide changes that possibly result in an aminoacidic substitution) or short in-frame insertions/deletions, which conversely do not affect full length protein,

Heterozygous *TTN*tv have been discovered in early 2000 s to be a leading cause of dilated cardiomyopathy (DCM) [3]. Numerous subsequent studies have rigorously confirmed the pathogenicity of *TTN*tv and their strong association with DCM [8]. When looking at clinically relevant regions across the gene, a recent study on more than 74,000 individuals that underwent genetic testing because of cardiomyopathy or arrhythmias, showed that *TTN*tv in all titin bands were significantly enriched in cases compared with controls [17]. 

However, the interpretation of *TTN*tv is complicated by a significant observation: these variants are also found in a notable proportion of seemingly healthy individuals, ranging from approximately 0.5% to 3% in community cohorts or biobanks [14, 18, 19]. This presence of *TTN*tv in asymptomatic individuals presents substantial challenges for genetic counseling and clinical decision-making. Furthermore, it complicates the determination of variant penetrance, which refers to the proportion of individuals with a particular genotype who express the associated phenotype. A potential way of discriminating is that *TTN*tv appear to be randomly distributed across the entire titin molecule in the general population, unlike in affected patients where they are enriched in specific regions, across all bands of the protein [20]. Moreover, while most individuals with *TTN*tv will not develop overt DCM, these variants are not necessarily phenotypically silent and may lead to slight increases in heart size, reduced contractility, and mild eccentric remodeling as shown by Ware et al. [21] These findings highlight a spectrum of clinical manifestations associated with *TTN*tv, ranging from asymptomatic carrier status to severe DCM, making careful follow-up and monitoring of individuals with these variants essential.

If *TTN*tv variant interpretation is challenging, data on missense variants shows that their prevalence in patient cohorts is almost undistinguishable from that in the general population. For example, a large study that analyzed over 60,000 ExAC individuals (Exome Aggregation Consortium) and 123,136 gnomAD database individuals found that rare heterozygous *TTN* missense variants predicted to be deleterious were observed in 5.7% of the gnomAD cohort [22]. Crucially, there was no significant enrichment of these variants in DCM patients compared to the general population. This lack of enrichment makes it exceedingly difficult to establish a direct causal link between most *TTN* missense variants and DCM.

While some researchers have proposed a potential modifier effect of *TTN* missense variants when co-inherited with other DCM-causing gene variants, such as those in the *LMNA* gene, this hypothesis is currently based on a very limited number of reported cases [23]. Therefore, while a modifier effect cannot be excluded, the evidence supporting it is still limited. Despite a few isolated reports of *TTN* missense variants being associated with disease [24], the vast majority of *TTN* missense variants encountered in clinical practice remain uninterpretable regarding their pathogenicity. Consequently, in the absence of compelling evidence to the contrary, these variants should generally be classified as “likely benign” in a clinical diagnostic workflow. This cautious approach prevents misdiagnosis and avoids unnecessary anxiety or interventions for individuals carrying these common genetic variations.

In conclusion, the current evidence strongly supports that *TTN*tv located in constitutively expressed exons (those with a PSI greater than 90%) can be considered disease associated. For these specific variants, genetic screening of family members is warranted, as recommended by professional guidelines such as those from the European Society of Cardiology [1]. This targeted screening can help identify at-risk individuals within families and facilitate early intervention or monitoring. Conversely, *TTN*ntv, particularly missense variants, should generally not be considered disease-causing in a Mendelian fashion.

## Molecular Mechanisms Underlying TTN-CMP

Titin forms a fundamental scaffold within the sarcomere, spanning half the length of the sarcomere, linking the Z-disc to the M-line. The protein functions as a molecular spring critical for maintaining passive tension, elasticity, and biomechanical signaling in cardiomyocytes. Despite their prevalence, however, the mechanisms by which *TTN*tv provoke cardiac dysfunction are still debated. The central question revolves around whether the disease arises predominantly through haploinsufficiency—the reduction of the amount of functional full-length titin protein—or through the presence of truncated titin peptides acting as “poison peptides”. Recent human tissue analyses, patient-derived cardiomyocytes, and animal models have begun to disentangle these complex mechanisms.

### Haploinsufficiency: Deficiency of Full-Length Titin Drives Sarcomere Dysfunction

Multiple studies analyzing human myocardium from TTN-CMP patients have consistently observed a 20–25% reduction in wild-type titin protein levels [14, 25, 26] compared to non-failing hearts, despite attempts by the remaining allele to compensate by upregulating titin mRNA expression [25, 27]. This persistent titin deficit seems associated to sarcomere insufficiency, characterized by decrease functional sarcomeres, impaired contractile force generation, and reduced myocardial stiffness. Importantly, experiments using human induced pluripotent stem cell–derived cardiomyocytes (hiPSC-CMs) harbouring patient-specific or engineered TTNtvs confirm that restoring wild-type titin levels—either via gene editing (e.g., CRISPR-Cas9) or proteasome inhibition—partially rescues contractile dysfunction, as further discussed in the “Models” Sects [25, 28]. This evidence firmly suggested haploinsufficiency as the core defect in TTNtv-mediated cardiomyopathy.

### Truncated Titin Proteins: Integration, Aggregation, and Toxicity

While early studies hypothesized that truncated titin proteins were rapidly degraded and thus unlikely to contribute significantly to pathology, more recent investigations have questioned this view. Using patient-specific antibodies targeting unique truncated titin epitopes, researchers have demonstrated that truncated titin proteins can persist in cardiac tissue [27, 29] and, in some cases, also integrate physically into the sarcomere [26, 29]. These truncated forms span from the Z-disc region and extend toward the A-band, mimicking the spatial localization of full-length titin. Functional assays reveal that, under physiological stretch, truncated titin fragments elongate similarly to normal titin and contribute to passive tension, but they show weaker interactions with the thick filament. These observations support the “poison peptide” model whereby truncated titin fragments do not represent a loss of function but also exert a dominant-negative effect by destabilizing sarcomeric structure and force transmission. Furthermore, in several *TTNtv* hearts, truncated titin proteins were found to accumulate predominantly as intracellular aggregates rather than integrating into sarcomeres. This aggregation correlates with impaired protein quality control (PQC) networks, including ubiquitin-proteasome system (UPS) dysfunction and overwhelmed autophagy pathways, which together exacerbate proteotoxic stress and cellular damage [25]. The accumulation of truncated proteins and PQC failure may contribute to disease progression during aging. Pharmacological modulation of PQC pathways, including proteasome inhibition, shows potential to elevate wild-type titin levels and improve sarcomere function, although toxicity limits clinical translation [25]. 

### Exon-Specific Effects and mRNA Surveillance Mechanisms

The fate of *TTNtv* transcripts is strongly exon-dependent. Indeed, the position of TTN truncations strongly influences the efficiency of nonsense-mediated mRNA decay (NMD), a surveillance pathway that degrades premature termination codon-containing transcripts [14]. Notably, a single study showed that truncations in exon 327, the largest exon of *TTN*, frequently escape NMD, resulting in elevated levels of mutant transcripts and enhanced truncated protein production [30]. This differential decay may concur in explaining the particular enrichment of exon 327 *TTNtv* in the cohorts of patients with DCM, since truncated titin proteins derived from these transcripts may be particularly toxic.

Ribosome profiling and transcriptomic analyses in patient hearts confirm active translation of these mutant transcripts, supporting a mechanistic link between NMD evasion and production of harmful truncated proteins. In contrast, truncations in other TTN regions tend to be efficiently degraded, leading primarily to haploinsufficiency with minimal truncated protein accumulation [27]. 

Thus, we can speculate that haploinsufficiency and “poison peptide” mechanisms might co-exist in TTN-CMP with variable contribution to the disease based on the site of the mutation.

MOLECULAR FOCUSES: In vitro and animal non-human models of TTN CMP.

**Table 1:** In vitro study models of TTN-related disease

Gene	Model Type	Observations
***TTN*** **(2 A-band** ***TTNtv***, **1 missense mutation)** [31]	Patient-derived hiPSC-CMs from 3 different patients, EHTs/CMTs	Sarcomere insufficiency, impaired contractility, blunted β-adrenergic & mechanical stress response, altered signaling.
***TTN*** **(*TTNtv*) **[32]	Patient-derived hiPSC-CMs; 2D monolayers & 3D microtissues	Poor sarcomere organization, reduced inotropic/lusitropic response to isoproterenol, prolonged caffeine recovery, calcium-handling abnormalities.
***TTN*** **(A-band** ***TTNtv*) **[33]	Genome-edited hiPSC-CMs; 3D microtissues	Sarcomeric proteinopathy, Na⁺ channel dysfunction, contractile defects; preserved core contractile machinery. Sarcomere modulators improved function.
***TTN*** **(Z- & A-band truncations) **[10]	CRISPR-edited hiPSC-EHTs (Z − and A − models)	Cronos titin partially rescues sarcomere formation in Z-band truncation; A-band truncation abolishes contraction.
***TTN*** **(A-band** ***TTNtv*)** [28]	Patient-derived hiPSC CMs (heterozygous *TTNtv*), genome-edited CRISPRa dCas9-VPR model; 3D microtissues	CRISPRa restored *TTN* protein levels and contractility despite increased truncated *TTN*. Rescued sarcomere content and normalized transcriptome. Demonstrated haploinsufficiency as major mechanism. Enabled cardiomyocyte-specific activation via enhancer targeting.
***RBM20*** **(ASO-mediated knockdown)** [14]	Human EHTs from control hiPSC-CMs + RBM20 ASOs	*RBM20* knockdown slowed relaxation, improved diastolic function in vitro. *TTN* isoform expression shifted toward more compliant forms, suggesting therapeutic benefit.
***RBM20*** **(p.Ser635Ala) **[34]	Patient-derived hiPSC-CMs (2 clones each), EHM	Severe DCM phenotype; sarcomere disarray, prolonged action potentials, abnormal calcium transients. *TTN* mis-splicing confirmed as a major consequence.
***RBM20*** **(missense/multiple variants) **[35]	Genetically edited hiPSC-CMs (e.g., R636S, P633L); 2D models	*RBM20* mutations cause defective splicing, impaired calcium handling and contractility; ATRA treatment restores *RBM20* and function. *TTN* mis-splicing is a central driver of disease phenotype.

Cardiac contractility is a key component of heart function and is closely associated with the progression of dilated cardiomyopathy [33]. Recent advances in various 3D models of induced pluripotent stem cell-derived cardiomyocytes (iPSC-CMs) [36, 37] have enabled more accurate modeling of sarcomere organization, allowing for quantitative assessment of force production and relaxation dynamics. Table 1 summarizes key studies employing 3D iPSC-CM models harboring titin truncating variants, highlighting common DCM-related phenotypes such as pathogenic proteinopathy, sarcomeric insufficiency, and structural abnormalities. *TTNtv* iPSC-CMs were either patient-derived [28, 31, 32] or generated via CRISPR-Cas9 gene editing [10, 33]. Across engineered heart tissue (EHT), cardiac microtissue (CMT), and other 3D platforms, reduced peak contractile force and abbreviated contraction–relaxation cycles have been consistently observed [31–33]. 

**Table 2:** Titin mouse models

Mouse Model	Genetic Modification	Phenotype & Key Features	Comments
mdm [38]	Spontaneous mutation causing titin dysfunction in skeletal muscle	Most studied titin model in skeletal muscle; severe myositis and dystrophy; used to characterize titin physiology outside the heart	Classic skeletal muscle model
N2B-KO [39]	Deletion of N2B-specific exon (human exon 49)	Smaller hearts but normal ejection volume; increased diastolic wall stress and dysfunction; cardiomyocytes have shortened sarcomere length and higher passive tension	Model of diastolic dysfunction without overt systolic failure
IG-KO [40]	Deletion of 9 Ig-like domains in proximal I-band (783 aa deleted)	Increased diastolic stiffness and cardiac hypertrophy resembling HFpEF; kyphosis; smaller soleus and diaphragm muscles; altered PEVK splicing in soleus muscle leading to smaller, stiffer isoforms	Demonstrates titin role in stiffness and muscle plasticity
TtnΔ112–158 [41]	Deletion of 47 PEVK exons (PEVK segment reduction)	Initially increased passive stiffness in soleus muscle; compensatory longitudinal hypertrophy with ~ 30% more sarcomeres; reduced body weight due to high energy cost of compensation	Highlights adaptive hypertrophy to maintain contractility
TtnΔ219–225 (PEVK-KO) [42]	Deletion of 7 constitutive PEVK exons in cardiac N2B isoform	Increased passive tension and diastolic dysfunction; cardiac hypertrophy with increased sarcomeres in series and parallel; upregulated FHL proteins; unexpected skeletal muscle stiffness increase	Links PEVK elasticity to active/passive mechanics and muscle plasticity
TtnΔIAjxn [43]	Deletion of 14 Ig-like and fibronectin III domains at I-band/A-band junction (exons 251–269)	Left ventricular hypertrophy and exercise intolerance; titin attachment shifted ~ 65 nm from Z-disc; resembles HFpEF phenotype	Shows importance of titin domain position in sarcomere mechanics
FINmaj [44]	11-bp insertion/deletion in last exon, altering M10 Ig domain	Heterozygotes develop mild late-onset myopathy (~ 9 months); homozygotes show early severe dystrophy, dilated cardiomyopathy, heart fibrosis, LV dysfunction	Finnish founder mutation modeling tibial muscular dystrophy
ΔMex5 [45]	Homozygous deletion of exon 363 encoding insertion sequence 7 (is7)	Causes dystrophic changes in aerobic skeletal muscles and dilated cardiomyopathy; disrupts titin scaffolding function, affects protein interactions	Highlights exon-specific role in cardiac and skeletal muscle function
TEV mouse model [46]	HaloTag-TEV cassette inserted in titin, allowing TEV protease cleavage	Controlled titin cleavage causes sarcomere disassembly without killing cells; no induced cardiomyocyte proliferation; acute cardiomyocyte detachment and fibrosis upon cleavage	Novel tool to dissect titin mechanics and signaling in vivo.

As show in Table 2, so far all the available mouse model were based on KO the gene or in introduction of mutations. The recently developed TEV mouse model, featuring a HaloTag-TEV cassette inserted into titin, introduces a groundbreaking genetic tool for precise, site-specific cleavage of titin in living muscle tissue [46]. This model enables targeted disruption of titin’s mechanical continuity without removing protein fragments or killing cardiomyocytes, offering a live, controlled system to study sarcomere disassembly and titin’s functional roles in vivo. In several recent studies it emerges that titin cleavage triggers sarcomere disassembly and acute remodeling (e.g., fibroblast activation, interstitial fibrosis) without immediate cell death. ^48^ This surprising information is currently helping in dissecting the titin important dual role as a structural and mechano-signaling molecule.

**Fig. 2:**
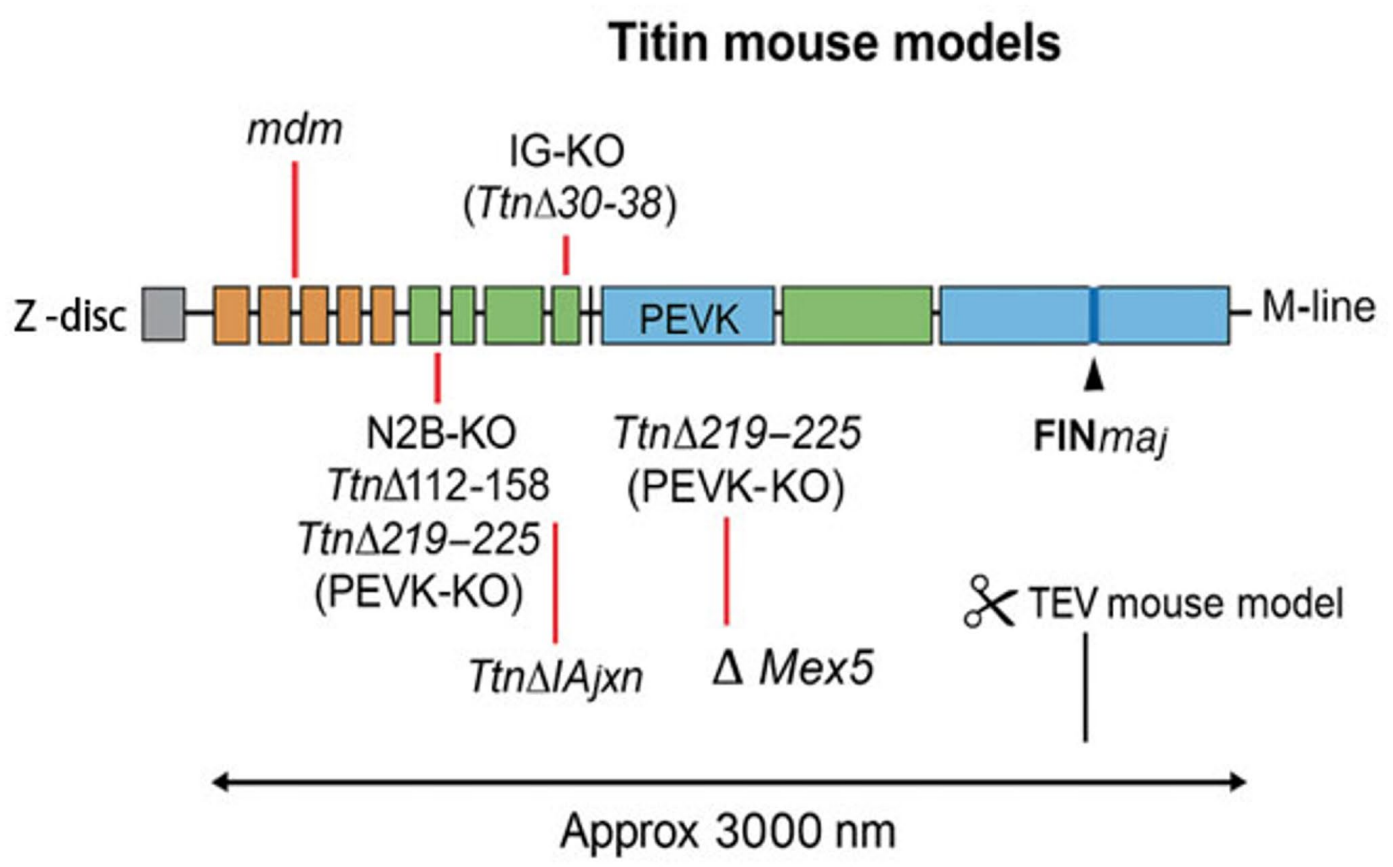
Titin mouse models according to the site of TTNtv.*Figure created using ChatGPT image generation (OpenAI)*,* August 2025. User retains full rights under OpenAI Terms of Use*

The TEV model decouples sarcomere integrity from cell survival, showing that mechanical disruption of titin is a potent driver of cardiac remodeling but not sufficient to reinitiate the cardiomyocyte cell cycle [48]. The TEV system thus offers a powerful platform to explore combinatorial strategies for cardiac regeneration by pairing sarcomere disassembly with mitogenic or signaling cues (Fig. 2).

#### Clinics

##### Genotype-Phenotype Association

Titin cardiomyopathy encompasses a broad clinical spectrum of diseases, in terms of presentation, morbidity and course, with a common genetic background tied to *TTN*tv [3]. 

*TTN*-CMP most commonly presents as DCM, a condition defined by varying degrees of left or biventricular dilation and systolic dysfunction in the absence of identifiable causes of volume or pressure overload [1]. In particular, among patients with DCM, TTN-CMP represents the most common genetic aetiology, responsible for approximately 18% of all DCM cases and up to 25% of genetically determined forms [3]. 

Recent studies have identified *TTN*tv in patients with Non-Dilated Left Ventricular Cardiomyopathy (NDLVC) [49]. These cases might also represent early-stage DCM detected through earlier diagnosis or family screening. The rate of progression among relatives of TTN-CMP patients is still under investigation, since penetrance studies are strongly influenced by population biases and time of follow up: a recent report by Del Mestre et al., found a global disease penetrance among adult carriers, identified by family screening, of 62% in about 10 years, [51]. Wheather timely medical intervention in relatives can slow this process remains still unknown. Further large ongoing studies will provide more definite data.

Isolated case reports and small series have suggested a possible association between *TTN* missense variants and arrhythmogenic right ventricular cardiomyopathy (ARVC) [51, 52], hypertrophic cardiomyopathy (HCM) [53, 54] and restrictive cardiomyopathy [55]. These associations remain uncertain due to insufficient evidence for definitive pathogenicity. Notably, missense *TTN* variants have been more consistently linked to skeletal muscle myopathies without necessary cardiac involvement [56]. 

## Clinical Presentation

The clinical presentation of *TTN*-CMP has evolved in recent years, largely due to advances in early diagnosis and family-based genetic screening.

The first cohort of *TTN*-CMP described in 2012 by *Herman et al.* [3] featured 67 *TTN*tv patients, mainly male individuals (71%) of Caucasian origin (90%), 31% with family history of heart failure without prior genetic investigation, and clinical presentation at a men age of 49 years old. Mean LVEF at baseline was 30–35%.

Eight years after, a large cohort study of *TTN*-CMP patients was published by Akhtar et al. [8], comprehensive of 537 individuals, among which 317 probands, 61% male, with a mean age at presentation of 45 years old for males and 49 years old for females. 69% had any degree LV dysfunction at baseline and among the whole population 28% presented with heart failure and NYHA 3 or 4. [8]

### Currently, In Probands, Heart Failure Represents the More Common Setting of First Presentation

In some cases, the presence of EKG alterations may prompt an elective cardiologic visit which will uncover the condition. Features such as abnormal T-wave inversion in precordial leads and low voltage QRS in peripheral leads have been respectively described in 40% and 12% of the patients with overt disease, while the presence of LBBB is uncommon in *TTN* cardiomyopathy [8, 57]. 

Subsequent diagnostic expansion has broadened recognition of *TTN*-CMP to include younger, often asymptomatic individuals identified through family screening, as well as older patients whose more subtle phenotypes may have previously gone unrecognized.

Recent studies have shown that the frequency of *TTN*tv in DCM with an onset during adolescence [58] is comparable to that observed in the adult population with DCM, weather is relatively higher in late onset DCM patients (after the age of 60 years) [59]. In contrast, *TTN*tv are rarely found in patients diagnosed with DCM in neonatal- and childhood [58]. 

The observation that the frequency of *TTN*tv is relatively increasing across patients with child-, adult-, and late-onset dilated DCM despite the considerable heterogeneity in disease presentation and progression, likely reflects the significant role of environmental modifying factors and the different individual genetic background (beyond *TTN*tv) in shaping clinical phenotype.

Indeed, the manifestation and progression of *TTN*-CMP is significantly influenced by comorbidities, environmental exposures, and behavioral factors. In line with the “second-hit” hypothesis [60], triggers such as alcohol consumption, chemotherapy, pregnancy, and the development of arrhythmias, particularly atrial fibrillation and other supraventricular arrhythmias, can precipitate adverse cardiac remodeling when not appropriately managed.

In many cases, these stressors precipitate the first clinical manifestation of HF in individuals carrying *TTN*tv, ultimately leading to diagnosis. Notably, in some single-centre series, up to 70% of *TTN*-CMP patients are reported to have at least one secondary risk factor contributing to the development of overt cardiomyopathy [60]. 

This accumulating evidence supports the recommendation to extend genetic testing to patients who develop DCM in the context of known cardiotoxic exposures, even in the absence of a family history [1]. In summary, clinicians should be aware that patients with DCM and carrier of TTNtv needs to be localized along a spectrum between two extremes: at one end, cases where TTNtv behaves largely as a bystander within a complex multifactorial etiological context; at the other, purely mendelian, familial forms in which TTNtv represent the primary cause of cardiac disease, allowing for meaningful genotype – phenotype prognostic, and therapeutic, correlations.

## Natural History

Following first diagnosis, the clinical course of *TTN*-CMP is notably heterogeneous. Compared to other forms of DCM, especially genetically determined subtypes, *TTN*-CMP is associated with higher rates of left ventricular reverse remodeling (LVRR) and lower incidence of adverse events during follow-up.

In the cohort reported by Akhtar et al. [8], LVRR occurred in 69% of patients with baseline LV dysfunction, with 54% achieving complete recovery of LVEF. Similarly, Escobar-López et al. [61] analyzed outcomes in DCM patients stratified by genetic findings and found that among those with pathogenic variants in established DCM-related genes, *TTN*tv were associated with the highest LVRR rate (56%) and a lower incidence of HF and arrhythmic events. After a median follow-up of 4.04 years, the cumulative incidence of major adverse cardiac events (MACE) in the *TTN*tv group was 22%, significantly lower than the overall cohort average of 32%. In line with these findings, the Akhtar cohort reported a 14.2% event rate over a mean follow-up of 49 months. Of these, 68% were due to end-stage HF and 32% to malignant ventricular arrhythmias (MVAs) [8].

*TTN*tv is independently associated with higher risk of developing ventricular and supraventricular arrhythmias [62]. Atrial fibrillation can be found in up to 30% of patients with *TTN*-CMP [8] and it is worth noting that 30% of those patients will later also develop LV dysfunction [63]. 

Premature ventricular complexes (PVCs) and non-sustained ventricular tachycardia (NSVT) are observed in up to 49% of patients, while sustained ventricular arrhythmias occur in 2–9%, with higher prevalence among those with advanced LV dysfunction [8]. 

Compared to cardiomyopathies caused by variants in *DSP*, *PKP2*, *FLNC*, and *LMNA* genes, *TTN*-CMP and sarcomeric gene-related cardiomyopathies tend to exhibit a similar burden of HF events but a generally lower arrhythmogenic profile [64]. Yet, the risk of MVAs during follow-up is still significant, particularly in the presence of environmental triggers [65]. 

The higher rates of LVRR and relatively lower rates of arrhythmias, combined with the relatively high prevalence of *TTN*tv in the general population, have led some in the cardiology community to refer to *TTN*-CMP as a “benign cardiomyopathy” [66]. Nonetheless, referring to *TTN*-CMP as a ‘benign cardiomyopathy’ may risk overlooking its potential for serious outcomes in selected patients.

## Risk Stratification

Cardiologists face many challenges in managing *TTN*-CMP, the most relevant being accurate risk stratification for major ventricular arrhythmias and heart failure events as well as predicting treatment response.

Indeed, current stratification tools are limited in their reliability and predictive value. The most predictive parameter currently available for clinical stratification is represented by LV dysfunction, since major cardiovascular events strictly correlate with lower LVEF [8]. In the Akhtar population above illustrated, all end point events occurred in patients with LVD at baseline or developed over follow-up.

Right ventricle might be involved in more aggressive forms of the condition, but a clear prognostic role of this finding has still to be investigated.

Arrhythmic burden itself is another feature investigated in recent studies for its prognostic relevance in *TTN*-CMP. Notably, Ebert and colleagues demonstrated in a small series that *TTN*-CMP patients presenting with sustained monomorphic ventricular tachycardia (SMVT) exhibited rapid progression to HF and death over a 5-year follow-up, with outcomes comparable to those seen in *LMNA*-related cardiomyopathy. In contrast, NSVT and PVCs were not associated with disease progression or worse outcomes [67]. 

Increasing use of CMR in clinical practice has proven to be a valuable tool for in vivo characterization of myocardial disease. In DCM patients, the presence of areas of interstitial or replacement fibrosis, identified as late gadolinium enhancement (LGE) or T1 mapping increase, have been consistently associated with worse outcomes due to arrhythmic burden and poor response to therapy, independently of *TTN* status [62]. In the CMR-subjected DCM population of Tayal et al., *TTN*-CMP showed 31% prevalence of LGE, similar to other DCMs for quantity and distribution, but it also showed thinner LV walls and lower LV mass.

Genetic data have so far proven difficult to integrate into outcome prediction for *TTN*-CMP. Previously, position of the truncating mutation was thought to be associated with different degrees of severity, and specifically *TTN*tv located more distally (near the A-band end) were proposed to lead to a more-severe phenotype [14]. However, the largest available study to date did not identify significant differences in prognosis based on the location of truncating variants across different *TTN* domains [8]. It should be considered, however, that nowadays several biases are still potentially limiting our knowledge on TTN-CMP genetics. In the past, this gene was not systematically screened in non-dilated CMP forms, neither in suspected channelopathies [68]. Furthermore, variants outside A-band have not been consistently considered pathogenic. New, unbiased studies are needed to definitely assess if the site in which the “tv” occurs may influence phenotype and prognosis.

Male sex is associated with higher penetrance and earlier clinical presentation in the broad class of DCM [69]. This was also confirmed in the context of *TTN*-CMP in a recent study involving a large cohort of individuals with *TTN* truncating variants which further identified male sex as an independent prognostic factor [8]. 

Finally, exercise have proven to be deleterious in favouring the progression of the disease in ARVC, deeming intense physical activity as unsuggested. In the case of *TTN*-CMP high level activity has not shown the same behaviour; indeed, a recent study showed that intense physical activity among *TTN*-CMP patients did not increase the rate of LV dysfunction and it did not increase the rate of adverse cardiovascular events [70].

## Management

Clinical management of *TTN*-CMP targets the interplay between the genetic background, the environment, and the phenotype of each patient. Interventions rely mainly on the treatment of heart failure and prevention of LV function deterioration.

According to cardiomyopathy and heart failure guidelines, in the presence of overt LV dysfunction, drug therapy consisting of beta-blockers, ACE inhibitors, MRA and SGLT2i should be administered and titrated [1, 71]. Achievement rates of optimal medical therapy (OMT) are generally high in both DCM and *TTN*-CMP cohorts [8, 72]. 

In patients who experience LVRR, continuation of therapy is recommended unless adverse effects or intolerance develop [1]. The role of therapy de-escalation following normalization of cardiac function remains unclear and is an area of ongoing investigation.

In *TTN*-CMP arrhythmias impose a substantial burden, contributing to low quality of life, influencing disease onset and progression, and serving as a significant cause of morbidity and mortality. AF generally presents at a younger age and has a higher tendency to progress to persistent or permanent AF. However, in a small cohort of *TTN*-CMP patients, ablation success rates resulted to be comparable with the ones of the general population [73].

In the context of ventricular arrhythmias, the arrhythmogenic substrate typically involves the basal septum and perivalvular regions. While catheter ablation may be considered for ventricular tachycardia (VT), long-term outcomes are often limited due to high recurrence rates, likely attributable to the intramural location of VT circuits [74]. 

ICD implantation is indicated in patients with persistent severe LV dysfunction despite OMT, as well as for secondary prevention in those who have survived a sudden cardiac death (SCD) event.

In the absence of clinical heart failure, *TTN*tv carriers do not have a current indication for pharmacologic therapy. Although earlier trials attempted pre-emptive treatment with canonical heart failure drugs in genotype-positive phenotype-negative relatives of DCM patients [75, 76], such strategies obtained heterogeneous results. However, no study has ever been tested specifically in *TTN*tv patients. Currently, in the presence of a known *TTN*tv background, clinical management aims at DCM prevention by controlling exposure to known triggers [60, 77].

Ongoing research is currently testing both old and newly discovered drugs based on the proposed physiology behind DCM, specifically *TTN*-CMP.

Omecamtiv mecarbil and Danicamtiv are myosin activators that produce a positive inotropic effect by enhancing ATP release from myosin filaments, thereby stabilizing cross-bridges in the sarcomere. In a mouse model of sarcomeric dilated cardiomyopathy (DCM) carrying a *TPM1* variant, omecamtiv mecarbil was shown to increase calcium sensitivity and improve isolated myofilament contractility [78]. However, in the broader population of heart failure patients with left ventricular dysfunction (LVD), omecamtiv mecarbil failed to demonstrate significant improvements in either mortality or functional outcomes. An ongoing trial testing Danicamtiv in a TTN-CMP population as well as further studies focusing on DCM patients, will help clarify the potential therapeutic role of myosin activators in these subgroups.

Emerging evidence suggests that altered sarcomere strain in titin-related cardiomyopathy (*TTN*-CMP) may drive a pathological shift in myosin head conformation from the super-relaxed (SRX) state to the disordered relaxed (DRX) state [7]. Based on this hypothesis, there is a rationale for exploring the use of myosin inhibitors (mavacamten, aficamten) beyond their current indications in hypertrophic cardiomyopathy (HCM) as potential modulators of sarcomeric kinetics and energetics in *TTN*-CMP.

In the context of tailored therapy, gene therapy strategies have been evaluated for the treatment of *TTN*-CMP in preclinical studies. Due to the large size of the *TTN* gene and increasing evidence supporting a dominant-negative effect of truncated *TTN* proteins, replacement therapy alone would not be sufficient.

More targeted approaches, including allele-specific silencing, could selectively reduce truncated titin production while preserving wild-type expression. A single study in patients’ derived iPSC and ki murine models, showed positive results of antisense oligonucleotides (AON) mediated exon skipping in preventing the development of DCM. ^80^ Additionally, therapeutics aimed at modulating titin elasticity or preventing proteotoxic aggregate formation represent exciting frontiers.

## Conclusions

Titin cardiomyopathy remains a frequent cause of morbidity and mortality, particularly among relatively young individuals. While genetic studies have elucidated many key aspects of the disease, the variable penetrance and wide spectrum of clinical severity highlight our still incomplete understanding of this condition. Current clinical characterization does not allow for efficient patient stratification, and management strategies are largely based on general heart failure guidelines, therefore identifying patients only in advanced stages of the disease. Advances in cellular and animal models have deepened our understanding of titin’s structural and signaling roles, as well as the effects of its mutations on the sarcomere, cardiomyocytes, and overall heart function. Building on these insights, future research should investigate how specific mutations differentially affect sarcomere biomechanics, protein interactions, and signaling pathways. Such efforts will be crucial for refining personalized treatment strategies for TTN-related cardiomyopathy.

## Data Availability

No datasets were generated or analysed during the current study.

## Abbreviations

AF: atrial fibrillation
CMR: cardiac magnetic resonance
CMT: cardiac microtissue
DCM: dilated cardiomyopathy
HF: heart failure
hiPSC-CMs: human induced pluripotent stem cell–derived cardiomyocytes
ICD: implantable cardiac defibrillator
LGE: late gadolinium enhancement
LV: left ventricle
LVD: left ventricular dysfunction
LVEF: left ventricular ejection fraction
LVRR: left ventricular reverse remodelling
MACE: major adverse cardiac events
MVA: major ventricular arrhythmias
NMD: nonsense-mediated decay
NSVT: non-sustained ventricular tachycardia
PQC: protein quality control
PSI: percentage spliced-in
SMVT: sustained monomorphic ventricular tachycardia
*TTN*: Titin (gene)
*TTN*-CMP: Titin induced cardiomyopathy
*TTN*tv: Titin truncating variant
*TTN*ntv: Titin non truncating variant
UPS: ubiquitin-proteasome system

## References

[1] Arbelo E, Protonotarios A, Gimeno JR, et al. 2023 ESC guidelines for the management of cardiomyopathies: developed by the task force on the management of cardiomyopathies of the European society of cardiology (ESC). Eur Heart J. 2023;44(37):3503–626. 10.1093/eurheartj/ehad194. 37622657 10.1093/eurheartj/ehad194

[2] Gigli M, Begay RL, Morea G, et al. A review of the giant protein Titin in clinical molecular diagnostics of cardiomyopathies. Front Cardiovasc Med. 2016;3. 10.3389/fcvm.2016.00021. 10.3389/fcvm.2016.00021 PMC4954824 27493940

[3] Herman DS, Lam L, Taylor MRG, et al. Truncations of Titin causing dilated cardiomyopathy. N Engl J Med. 2012;366(7):619–28. 10.1056/NEJMoa1110186. 22335739 10.1056/NEJMoa1110186 PMC3660031

[4] Tharp CA, Haywood ME, Sbaizero O, Taylor MRG, Mestroni L. The giant protein titin’s role in cardiomyopathy: Genetic, Transcriptional, and Post-translational modifications of TTN and their contribution to cardiac disease. Front Physiol. 2019;10:1436. 10.3389/fphys.2019.01436. 31849696 10.3389/fphys.2019.01436 PMC6892752

[5] Akinrinade O, Koskenvuo JW, Alastalo TP. Prevalence of Titin truncating variants in general population. PLoS ONE. 2015;10(12):e0145284. 10.1371/journal.pone.0145284. 26701604 10.1371/journal.pone.0145284 PMC4689403

[6] Stroeks SLVM, van Beek D, Adriaens ME, Verdonschot JAJ. Titin allelic expression and protein processing pathways in Early-Stage dilated cardiomyopathy patients with truncating Titin variants. Circ Genomic Precis Med. 2023;16(1):e003901. 10.1161/CIRCGEN.122.003901. 10.1161/CIRCGEN.122.003901 36716182

[7] Granzier HL, Labeit S. Discovery of Titin and its role in heart function and disease. Circ Res. 2025;136(1):135–57. 10.1161/CIRCRESAHA.124.323051. 39745989 10.1161/CIRCRESAHA.124.323051

[8] Akhtar MM, Lorenzini M, Cicerchia M, et al. Clinical phenotypes and prognosis of dilated cardiomyopathy caused by truncating variants in the TTN gene. Circ Heart Fail. 2020;13(10):e006832. 10.1161/CIRCHEARTFAILURE.119.006832. 32964742 10.1161/CIRCHEARTFAILURE.119.006832

[9] Huebsch KA, Kudryashova E, Wooley CM, et al. Mdm muscular dystrophy: interactions with Calpain 3 and a novel functional role for titin’s N2A domain. Hum Mol Genet. 2005;14(19):2801–11. 10.1093/hmg/ddi313. 16115818 10.1093/hmg/ddi313 PMC1350399

[10] Zaunbrecher RJ, Abel AN, Beussman K, et al. Cronos Titin is expressed in human cardiomyocytes and necessary for normal sarcomere function. Circulation. 2019;140(20):1647–60. 10.1161/CIRCULATIONAHA.119.039521. 31587567 10.1161/CIRCULATIONAHA.119.039521 PMC6911360

[11] Bang ML, Centner T, Fornoff F, et al. The complete gene sequence of Titin, expression of an Unusual ≈ 700-kDa Titin Isoform, and its interaction with obscurin identify a novel Z-Line to I-Band linking system. Circ Res. 2001;89(11):1065–72. 10.1161/hh2301.100981. 11717165 10.1161/hh2301.100981

[12] Labeit S, Kolmerer B. Titins: giant proteins in charge of muscle ultrastructure and elasticity. Science. 1995;270(5234):293–6. 10.1126/science.270.5234.293. 7569978 10.1126/science.270.5234.293

[13] LeWinter MM, Granzier HL. Titin is a major human disease gene. Circulation. 2013;127(8):938–44. 10.1161/CIRCULATIONAHA.112.139717. 23439446 10.1161/CIRCULATIONAHA.112.139717 PMC3594684

[14] Roberts AM, Ware JS, Herman DS, et al. Integrated allelic, transcriptional, and phenomic dissection of the cardiac effects of Titin truncations in health and disease. Sci Transl Med. 2015;7(270):270ra6. 10.1126/scitranslmed.3010134. 25589632 10.1126/scitranslmed.3010134 PMC4560092

[15] Guo W, Schafer S, Greaser ML, et al. RBM20, a gene for hereditary cardiomyopathy, regulates Titin splicing. Nat Med. 2012;18(5):766–73. 10.1038/nm.2693. 22466703 10.1038/nm.2693 PMC3569865

[16] Guo W, Zhu C, Yin Z, et al. Splicing factor RBM20 regulates transcriptional network of Titin associated and calcium handling genes in the heart. Int J Biol Sci. 2018;14(4):369–80. 10.7150/ijbs.24117. 29725258 10.7150/ijbs.24117 PMC5930469

[17] Vatta M, Regalado E, Parfenov M, et al. Analysis of TTN truncating variants in > 74 000 cases reveals new clinically relevant gene regions. Circ Genomic Precis Med. 2025;18(2):e004982. 10.1161/CIRCGEN.124.004982. 10.1161/CIRCGEN.124.004982 PMC11999099 39968638

[18] Schafer S, de Marvao A, Adami E, et al. Titin-truncating variants affect heart function in disease cohorts and the general population. Nat Genet. 2017;49(1):46–53. 10.1038/ng.3719. 27869827 10.1038/ng.3719 PMC5201198

[19] Mazzarotto F, Tayal U, Buchan RJ, et al. Reevaluating the genetic contribution of Monogenic dilated cardiomyopathy. Circulation. 2020;141(5):387–98. 10.1161/CIRCULATIONAHA.119.037661. 31983221 10.1161/CIRCULATIONAHA.119.037661 PMC7004454

[20] Linke WA. Titin gene and protein functions in passive and active muscle. Annu Rev Physiol. 2018;80:389–411. 10.1146/annurev-physiol-021317-121234. 29131758 10.1146/annurev-physiol-021317-121234

[21] Ware JS, Cook SA. Role of Titin in cardiomyopathy: from DNA variants to patient stratification. Nat Rev Cardiol. 2018;15(4):241–52. 10.1038/nrcardio.2017.190. 29238064 10.1038/nrcardio.2017.190

[22] Akinrinade O, Heliö T, Lekanne Deprez RH, et al. Relevance of Titin missense and Non-Frameshifting Insertions/Deletions variants in dilated cardiomyopathy. Sci Rep. 2019;9(1):4093. 10.1038/s41598-019-39911-x. 30858397 10.1038/s41598-019-39911-x PMC6412046

[23] Roncarati R, Viviani Anselmi C, Krawitz P, et al. Doubly heterozygous LMNA and TTN mutations revealed by exome sequencing in a severe form of dilated cardiomyopathy. Eur J Hum Genet EJHG. 2013;21(10):1105–11. 10.1038/ejhg.2013.16. 23463027 10.1038/ejhg.2013.16 PMC3778353

[24] Domínguez F, Lalaguna L, Martínez-Martín I, et al. Titin missense variants as a cause of Familial dilated cardiomyopathy. Circulation. 2023;147(22):1711–3. 10.1161/CIRCULATIONAHA.122.062833. 37253077 10.1161/CIRCULATIONAHA.122.062833

[25] Fomin A, Gärtner A, Cyganek L, et al. Truncated Titin proteins and Titin haploinsufficiency are targets for functional recovery in human cardiomyopathy due to TTN mutations. Sci Transl Med. 2021;13(618):eabd3079. 10.1126/scitranslmed.abd3079. 34731013 10.1126/scitranslmed.abd3079

[26] McAfee Q, Caporizzo MA, Uchida K et al. Truncated Titin protein in dilated cardiomyopathy incorporates into the sarcomere and transmits force. J Clin Invest 134(2):e170196. 10.1172/JCI170196 10.1172/JCI170196 PMC10786684 37943622

[27] Hinson JT, Campbell SG. TTN Truncation variants produce sarcomere-integrating proteins of uncertain functional significance. J Clin Invest. 2024;134(2):e175206. 10.1172/JCI175206. 38226618 10.1172/JCI175206 PMC10786689

[28] Ghahremani S, Kanwal A, Pettinato A, et al. CRISPR activation reverses haploinsufficiency and functional deficits caused by TTN Truncation variants. Circulation. 2024;149(16):1285–97. 10.1161/CIRCULATIONAHA.123.063972. 38235591 10.1161/CIRCULATIONAHA.123.063972 PMC11031707

[29] Kellermayer D, Tordai H, Kiss B et al. Truncated Titin is structurally integrated into the human dilated cardiomyopathic sarcomere. J Clin Invest 134(2):e169753. 10.1172/JCI169753 10.1172/JCI169753 PMC10763722 37962957

[30] Kim Y, Kim SW, Saul D et al. Regulation of sarcomere formation and function in the healthy heart requires a Titin intronic enhancer. J Clin Invest. 135(4):e183353. 10.1172/JCI183353 10.1172/JCI183353 PMC11827849 39688912

[31] Hinson J, Chopra A, Nafissi N, et al. HEART DISEASE. Titin mutations in iPS cells define sarcomere insufficiency as a cause of dilated cardiomyopathy. Science. 2015;349:982–6. 26315439 10.1126/science.aaa5458 PMC4618316

[32] Schick R, Mekies L, Shemer Y, Eisen B, Hallas T. others. Functional abnormalities in induced pluripotent stem Cell-derived cardiomyocytes generated from titin-mutated patients with dilated cardiomyopathy. PLoS ONE. 2018;13(10):e0205719. 10.1371/journal.pone.0205719. 30332462 10.1371/journal.pone.0205719 PMC6192629

[33] Huang G, Bisaria A, Wakefield DL, et al. Titin-truncating variants in HiPSC cardiomyocytes induce pathogenic proteinopathy and sarcomere defects with preserved core contractile machinery. Stem Cell Rep. 2023;18(1):220–36. 10.1016/j.stemcr.2022.11.008. 10.1016/j.stemcr.2022.11.008 PMC9860080 36525964

[34] Streckfuss-Bömeke K, Tiburcy M, Fomin A, et al. Severe DCM phenotype of patient harboring RBM20 mutation S635A can be modeled by patient-specific induced pluripotent stem cell-derived cardiomyocytes. J Mol Cell Cardiol. 2017;113:9–21. 10.1016/j.yjmcc.2017.09.008. 28941705 10.1016/j.yjmcc.2017.09.008

[35] Briganti F, Sun H, Wei W, et al. iPSC modeling of RBM20-Deficient DCM identifies upregulation of RBM20 as a therapeutic strategy. Cell Rep. 2020;32(10):108117. 10.1016/j.celrep.2020.108117. 32905764 10.1016/j.celrep.2020.108117 PMC8168789

[36] Thavandiran N, Dubois N, Mikryukov A, et al. Design and formulation of functional pluripotent stem cell-derived cardiac microtissues. Proc Natl Acad Sci U A. 2013;110(49):E4698–707. 10.1073/pnas.1311120110. 10.1073/pnas.1311120110 PMC3856835 24255110

[37] Mannhardt I, Breckwoldt K, Letuffe-Brenière D, et al. Human engineered heart tissue: analysis of contractile force. Stem Cell Rep. 2016;7(1):29–42. 10.1016/j.stemcr.2016.04.011. 10.1016/j.stemcr.2016.04.011 PMC4944531 27211213

[38] Garvey SM, Rajan C, Lerner AP, Frankel WN, Cox GA. The muscular dystrophy with myositis (mdm) mouse mutation disrupts a skeletal muscle-specific domain of Titin. Genomics. 2002;79(2):146–9. 11829483 10.1006/geno.2002.6685

[39] Radke MH, Peng J, Wu Y, et al. Targeted deletion of Titin N2B region leads to diastolic dysfunction and cardiac atrophy. Proc Natl Acad Sci U A. 2007;104(9):3444–9. 10.1073/pnas.0608543104 PMC1805563 17360664

[40] Chung CS, Hutchinson KR, Methawasin M, et al. Shortening of the elastic tandem Immunoglobulin segment of Titin leads to diastolic dysfunction. Circulation. 2013;128(1):19–28. 23709671 10.1161/CIRCULATIONAHA.112.001268 PMC3822017

[41] Brynnel A, Hernandez Y, Kiss B, et al. Downsizing the molecular spring of the giant protein Titin reveals that skeletal muscle Titin determines passive stiffness and drives longitudinal hypertrophy. Elife. 2018;7. 10.7554/eLife.40532. 10.7554/eLife.40532 PMC6300359 30565562

[42] Granzier HL, Radke MH, Peng J, et al. Truncation of titin’s elastic PEVK region leads to cardiomyopathy with diastolic dysfunction. Circ Res. 2009;105(6):557–64. 19679835 10.1161/CIRCRESAHA.109.200964 PMC2785004

[43] Granzier HL, Hutchinson KR, Tonino P, et al. Deleting Titin’s I-band/A-band junction reveals critical roles for Titin in Biomechanical sensing and cardiac function. Proc Natl Acad Sci U A. 2014;111(40):14589–94. 10.1073/pnas.1411493111 PMC4210014 25246556

[44] Charton K, Daniele N, Vihola A, et al. Removal of the Calpain 3 protease reverses the myopathology in a mouse model for titinopathies. Hum Mol Genet. 2010;19(23):4608–24. 20855473 10.1093/hmg/ddq388

[45] Charton K, Suel L, Henriques SF, et al. Exploiting the CRISPR/Cas9 system to study alternative splicing in vivo: application to Titin. Hum Mol Genet. 2016;25(20):4518–32. 28173117 10.1093/hmg/ddw280

[46] Silva-Rojas R, Vicente N, Gavilán-Herrera M, et al. Mechanically knocking out Titin reveals protein tension loss as a trigger of muscle disease. Nat Biomed Eng Published Online June. 2025;5. 10.1038/s41551-025-01403-x. 10.1038/s41551-025-01403-x 40473933

[47] López-Unzu MA, Pricolo MR, Silva-Rojas R et al. Titin cleavage is a driver of cardiomyocyte disengagement and reactive myocardial fibrosis. Published online April 22, 2025:2025.04.22.645683. 10.1101/2025.04.22.645683

[48] Pricolo MR, López-Unzu MA, Vicente N et al. Titin cleavage in living cardiomyocytes induces sarcomere disassembly but does not trigger cell proliferation. Published online April 22, 2025:2025.04.22.645658. 10.1101/2025.04.22.645658 10.1016/j.jbc.2026.113167 PMC13279373 42142587

[49] Castrichini M, De LA, De AG, et al. Magnetic resonance imaging characterization and clinical outcomes of dilated and arrhythmogenic left ventricular cardiomyopathies. JACC. 2024;83(19):1841–51. 10.1016/j.jacc.2024.02.041. 38719365 10.1016/j.jacc.2024.02.041 PMC12042165

[50] Del Mestre E, Paldino A, Pio Loco Detto Gava C, et al. Prediction and prognostic role of left ventricular systolic dysfunction in family screening for dilated cardiomyopathy and non-dilated left ventricular cardiomyopathy. Eur J Heart Fail Published Online April. 2025;13. 10.1002/ejhf.3657. 10.1002/ejhf.3657 PMC12803633 40222818

[51] Taylor M, Graw S, Sinagra G, et al. Genetic variation in Titin in arrhythmogenic right ventricular Cardiomyopathy–Overlap syndromes. Circulation. 2011;124(8):876–85. 10.1161/CIRCULATIONAHA.110.005405. 21810661 10.1161/CIRCULATIONAHA.110.005405 PMC3167235

[52] Brun F, Barnes CV, Sinagra G, et al. Titin and desmosomal genes in the natural history of arrhythmogenic right ventricular cardiomyopathy. J Med Genet. 2014;51(10):669–76. 10.1136/jmedgenet-2014-102591. 25157032 10.1136/jmedgenet-2014-102591 PMC4465780

[53] Satoh M, Takahashi M, Sakamoto T, Hiroe M, Marumo F, Kimura A. Structural analysis of the Titin gene in hypertrophic cardiomyopathy: identification of a novel disease gene. Biochem Biophys Res Commun. 1999;262(2):411–7. 10.1006/bbrc.1999.1221. 10462489 10.1006/bbrc.1999.1221

[54] Itoh-Satoh M, Hayashi T, Nishi H, et al. Titin mutations as the molecular basis for dilated cardiomyopathy. Biochem Biophys Res Commun. 2002;291(2):385–93. 10.1006/bbrc.2002.6448. 11846417 10.1006/bbrc.2002.6448

[55] Peled Y, Gramlich M, Yoskovitz G, et al. Titin mutation in Familial restrictive cardiomyopathy. Int J Cardiol. 2014;171(1):24–30. 10.1016/j.ijcard.2013.11.037. 24315344 10.1016/j.ijcard.2013.11.037

[56] Rees M, Nikoopour R, Fukuzawa A, et al. Making sense of missense variants in TTN-related congenital myopathies. Acta Neuropathol (Berl). 2021;141(3):431–53. 10.1007/s00401-020-02257-0. 33449170 10.1007/s00401-020-02257-0 PMC7882473

[57] Dal Ferro M, Paldino A, Gregorio C, et al. Impact of DCM-Causing genetic background on Long-Term response to cardiac resynchronization therapy. JACC Clin Electrophysiol. 2024;10(7 Pt 1):1455–64. 10.1016/j.jacep.2024.03.019. 38795101 10.1016/j.jacep.2024.03.019 PMC12044666

[58] Fatkin D, Lam L, Herman DS, et al. Titin truncating mutations: a rare cause of dilated cardiomyopathy in the young. Prog Pediatr Cardiol. 2016;40:41–5. 10.1016/j.ppedcard.2016.01.003.

[59] Cannatà A, Merlo M, Dal Ferro M, et al. Association of Titin variations with Late-Onset dilated cardiomyopathy. JAMA Cardiol. 2022;7(4):371–7. 10.1001/jamacardio.2021.5890. 35138330 10.1001/jamacardio.2021.5890 PMC8829739

[60] Giudicessi JR, Shrivastava S, Ackerman MJ, Pereira NL. Clinical impact of secondary risk factors in TTN-Mediated dilated cardiomyopathy. Circ Genomic Precis Med. 2021;14(2):e003240. 10.1161/CIRCGEN.120.003240. 10.1161/CIRCGEN.120.003240 PMC8284363 33866824

[61] Escobar-Lopez L, Ochoa JP, Mirelis JG, et al. Association of genetic variants with outcomes in patients with nonischemic dilated cardiomyopathy. J Am Coll Cardiol. 2021;78(17):1682–99. 10.1016/j.jacc.2021.08.039. 34674813 10.1016/j.jacc.2021.08.039

[62] Tayal U, Newsome S, Buchan R, et al. Truncating variants in Titin independently predict early arrhythmias in patients with dilated cardiomyopathy. J Am Coll Cardiol. 2017;69(19):2466–8. 10.1016/j.jacc.2017.03.530. 28494986 10.1016/j.jacc.2017.03.530 PMC5423712

[63] Schiabor Barrett KM, Cirulli ET, Bolze A, et al. Cardiomyopathy prevalence exceeds 30% in individuals with TTN variants and early atrial fibrillation. Genet Med Off J Am Coll Med Genet. 2023;25(4):100012. 10.1016/j.gim.2023.100012. 10.1016/j.gim.2023.100012 36637017

[64] Paldino A, Dal FM, Stolfo D, et al. Prognostic prediction of genotype vs phenotype in genetic cardiomyopathies. JACC. 2022;80(21):1981–94. 10.1016/j.jacc.2022.08.804. 36396199 10.1016/j.jacc.2022.08.804 PMC10754019

[65] Verdonschot JAJ, Hazebroek MR, Derks KWJ, et al. Titin cardiomyopathy leads to altered mitochondrial energetics, increased fibrosis and long-term life-threatening arrhythmias. Eur Heart J. 2018;39(10):864–73. 10.1093/eurheartj/ehx808. 29377983 10.1093/eurheartj/ehx808

[66] Jansweijer JA, Nieuwhof K, Russo F, et al. Truncating Titin mutations are associated with a mild and treatable form of dilated cardiomyopathy. Eur J Heart Fail. 2017;19(4):512–21. 10.1002/ejhf.673. 27813223 10.1002/ejhf.673

[67] Ebert M, de Riva M, Wijnmaalen AP et al. The relevance of the type of ventricular arrhythmia in titin-related dilated cardiomyopathy: a multicenter study. JACC Clin Electrophysiol. Published online February 21, 2025:S2405-500X(25)00066 – 0. 10.1016/j.jacep.2025.01.010 10.1016/j.jacep.2025.01.010 40088216

[68] Dellefave-Castillo LM, Cirino AL, Callis TE, et al. Assessment of the diagnostic yield of combined cardiomyopathy and arrhythmia genetic testing. JAMA Cardiol. 2022;7(9):966–74. 10.1001/jamacardio.2022.2455. 35947370 10.1001/jamacardio.2022.2455 PMC9366660

[69] Argirò A, Ho C, Day SM, et al. Sex-Related differences in genetic cardiomyopathies. J Am Heart Assoc. 2022;11(9):e024947. 10.1161/JAHA.121.024947. 35470690 10.1161/JAHA.121.024947 PMC9238595

[70] Savonitto G, Paldino A, Setti M, et al. Exercise intensity and cardiac disease development in carriers of Titin variants. Eur J Prev Cardiol Published Online Febr. 2025;25:zwaf094. 10.1093/eurjpc/zwaf094. 10.1093/eurjpc/zwaf094 39999024

[71] McDonagh TA, Metra M, Adamo M, et al. 2021 ESC guidelines for the diagnosis and treatment of acute and chronic heart failure: developed by the task force for the diagnosis and treatment of acute and chronic heart failure of the European society of cardiology (ESC) with the special contribution of the heart failure association (HFA) of the ESC. Eur Heart J. 2021;42(36):3599–726. 10.1093/eurheartj/ehab368. 34447992 10.1093/eurheartj/ehab368

[72] Hertzog T, Picard F, Bonnet G, Domingues-Dos-Santos P. Prevalence of early and late left ventricular reverse remodeling in dilated cardiomyopathy under optimal medical therapy. Arch Cardiovasc Dis Suppl. 2023;15(1):42–3. 10.1016/j.acvdsp.2022.10.076.

[73] Virk ZM, El -Harasis Majd A, Yoneda ZT, et al. Clinical characteristics and outcomes in patients with atrial fibrillation and pathogenic TTN variants. JACC Clin Electrophysiol. 2024;10(11):2445–57. 10.1016/j.jacep.2024.07.029. 39453294 10.1016/j.jacep.2024.07.029

[74] Enriquez A, Liang J, Smietana J, et al. Substrate characterization and outcomes of ventricular tachycardia ablation in TTN (Titin) cardiomyopathy. Circ Arrhythm Electrophysiol. 2021;14(9):e010006. 10.1161/CIRCEP.121.010006. 34315225 10.1161/CIRCEP.121.010006

[75] de Brouwer R, te Rijdt WP, Hoorntje ET, et al. A randomized controlled trial of eplerenone in asymptomatic phospholamban p.Arg14del carriers. Eur Heart J. 2023;44(40):4284–7. 10.1093/eurheartj/ehad292. 37210081 10.1093/eurheartj/ehad292 PMC10590125

[76] Allen HD, Flanigan KM, Thrush PT, et al. A Randomized, double-blind trial of Lisinopril and Losartan for the treatment of cardiomyopathy in Duchenne muscular dystrophy. PLoS Curr. 2013;5: 2bcb420024ea865. 10.1371/currents.md.2cc69a1dae4be7dfe2bcb420024ea865. 10.1371/currents.md.2cc69a1dae4be7dfe2bcb420024ea865 PMC3871420 24459612

[77] Verdonschot JAJ, Hazebroek MR, Ware JS, Prasad SK, Heymans SRB. Role of targeted therapy in dilated cardiomyopathy: the challenging road toward a personalized approach. J Am Heart Assoc. 2019;8(11):e012514. 10.1161/JAHA.119.012514. 31433726 10.1161/JAHA.119.012514 PMC6585365

[78] Utter MS, Ryba DM, Li BH, Wolska BM, Solaro RJ. Omecamtiv Mecarbil, a cardiac myosin activator, increases Ca2 + Sensitivity in myofilaments with a dilated cardiomyopathy mutant Tropomyosin E54K. J Cardiovasc Pharmacol. 2015;66(4):347–53. 10.1097/FJC.0000000000000286. 26065842 10.1097/FJC.0000000000000286 PMC5460532

[79] Gramlich M, Pane LS, Zhou Q, et al. Antisense-mediated exon skipping: a therapeutic strategy for titin-based dilated cardiomyopathy. EMBO Mol Med. 2015;7(5):562–76. 10.15252/emmm.201505047. 25759365 10.15252/emmm.201505047 PMC4492817

